# Supplementary material for: HIVprotI: an integrated web based platform for prediction and design of HIV proteins inhibitors
Source: J Cheminform. 2018 Mar 9;10:12. doi: 10.1186/s13321-018-0266-y (PMC5845081; doi:10.1186/s13321-018-0266-y)
Supplement: Supplementary file 2 — Additional file 2. Source code of HIVProtI web server. [file 13321_2018_266_MOESM2_ESM.zip › HIVprotI_Source-code/hivprotI/weka-3-6-6/doc/weka/core/matrix/package-summary.html]

weka.core.matrix


---


|  |  |  |
| --- | --- | --- |
| **Overview** \| **Package** \| Class \| **Tree** \| **Deprecated** \| **Index** \| **Help** \| **Weka's home** | |  |
| **PREV PACKAGE**   **NEXT PACKAGE** | **FRAMES**    **NO FRAMES**     **All Classes** |  |


---

## Package weka.core.matrix

| **Class Summary** | |
| --- | --- |
| **CholeskyDecomposition** | Cholesky Decomposition. |
| **DoubleVector** | A vector specialized on doubles. |
| **EigenvalueDecomposition** | Eigenvalues and eigenvectors of a real matrix. |
| **ExponentialFormat** |  |
| **FlexibleDecimalFormat** |  |
| **FloatingPointFormat** | Class for the format of floating point numbers |
| **IntVector** | A vector specialized on integers. |
| **LinearRegression** | Class for performing (ridged) linear regression. |
| **LUDecomposition** | LU Decomposition. |
| **Maths** | Utility class. |
| **Matrix** | Jama = Java Matrix class. |
| **QRDecomposition** | QR Decomposition. |
| **SingularValueDecomposition** | Singular Value Decomposition. |

---


|  |  |  |
| --- | --- | --- |
| **Overview** \| **Package** \| Class \| **Tree** \| **Deprecated** \| **Index** \| **Help** \| **Weka's home** | |  |
| **PREV PACKAGE**   **NEXT PACKAGE** | **FRAMES**    **NO FRAMES**     **All Classes** |  |


---
